# Supplementary material for: Effects of Allium mongolicum Regel and Its Flavonoids on Constipation
Source: Biomolecules. 2019 Dec 20;10(1):14. doi: 10.3390/biom10010014 (PMC7022811; doi:10.3390/biom10010014)
Supplement: Supplementary file 1 [file biomolecules-10-00014-s001.pdf]

biomolecules

MDPI

Supplementary Materials: The following are available online at www.mdpi.com/xxx/s1,

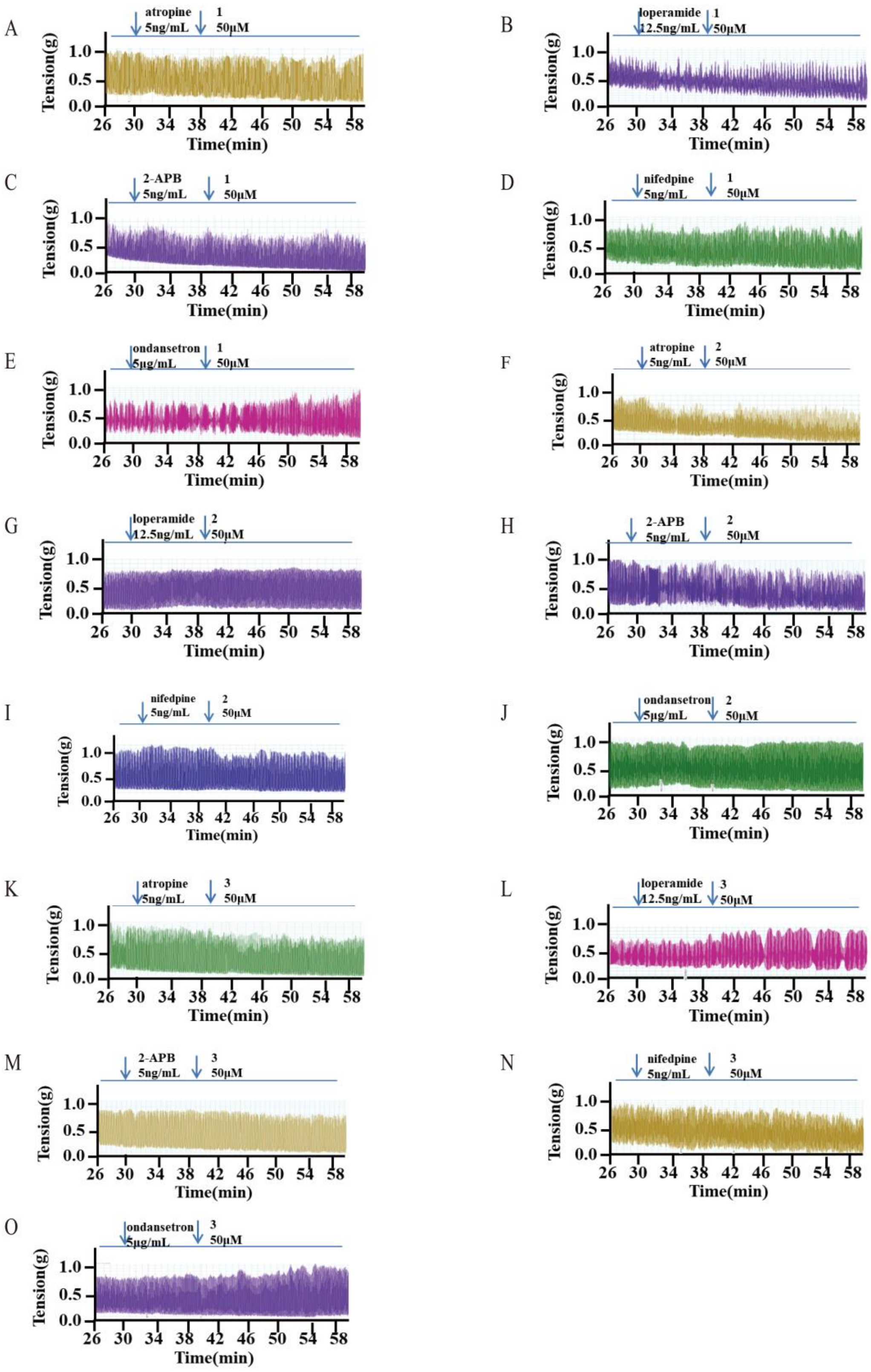

Figure S1: Intestinal contraction under AM flavonoids-treated with inhibitors. (A-O) Effects of 1 (A-E), 2 (F-J) and 3 (K-O) on spontaneous contractions of isolated intestinal contraction with different inhibitors.

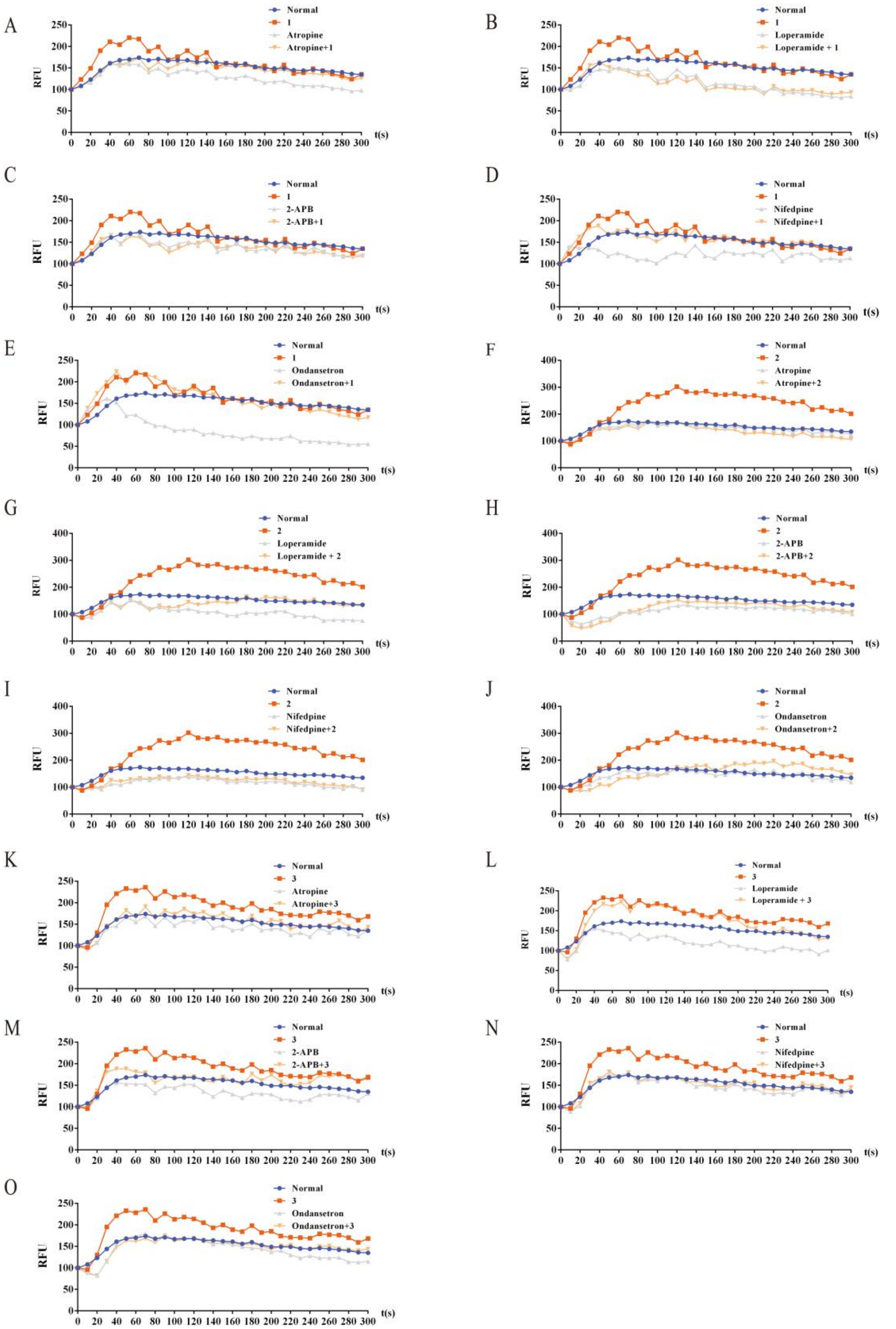

Figure S2: Calcium influx under AM flavonoids-treated with inhibitors. (A-O) Effects of 1 (A-E), 2 (F-J) and 3 (K-O) on isolated calcium influx of HCT116 with different inhibitors.

© 2019 by the authors. Submitted for possible open access publication under the terms and conditions of the Creative Commons Attribution (CC BY) license (http://creativecommons.org/licenses/by/4.0/).
